# Study on the relationship between hormone and Lp(a) in Chinese overweight/obese patients

**DOI:** 10.1186/s12902-022-01021-7

**Published:** 2022-05-16

**Authors:** Xiaona Chang, Nannan Bian, Xiaoyu Ding, Jinman Li, Yu An, Jiaxuan Wang, Jia Liu, Guang Wang

**Affiliations:** Department of Endocrinology, Beijing Chao-Yang Hospital, Capital Medical University, No. 8, Gongti South Road, Chaoyang District, Beijing, 100020 China

**Keywords:** Lp(a), Testosterone, Obesity, Regression analysis

## Abstract

**Background:**

Obesity is a risk factor for metabolic diseases and often influences hormone change. Lipoprotein (a) (Lp(a)) is associated with various metabolic diseases, but there are few studies on the relationship between Lp(a) and hormones in obese patients. This study investigated the the relationship between Lp(a) and hormones in Chinese overweight/obese people.

**Methods:**

A total of 410 overweight/obese patients (Body mass index (BMI) ≥ 25 kg/m^2^) were included and underwent sociodemographic data investigations and relevant clinical examinations. Lp(a) was analyzed by colorimetric enzymatic assays and hormone was measured with chemiluminescence immunoassay method. According to Lp(a) levels, they were categorized into 3 groups: the lower Lp(a) group (Lp(a) levels < 30 mg/dl), the moderate Lp(a) group (Lp(a) levels between 30 mg/dl and 120 mg/dl) and the higher Lp(a) group (Lp(a) levels > 120 mg/dl). The differences of hormone levels among the three groups were compared and the relationship between Lp(a) and hormones was analyzed by Spearman’s rank correlation.

**Results:**

The higher Lp(a) group had significantly lower testosterone (TES) levels compared with the lower and moderate Lp(a) groups in the case of gender, age and BMI matching. Lp(a) concentration was negatively correlated with TES levels in all participants and the negative association between Lp(a) and TES levels was also observed when the analysis was stratified by gender. Additionally, the TES was statistically related with Lp(a) levels in the multiple linear regression model (95% confidence interval: − 0.451 to − 0.079).

**Conclusions:**

TES levels was negatively associated with Lp(a) levels in Chinese overweight/obese patients.

**Supplementary Information:**

The online version contains supplementary material available at 10.1186/s12902-022-01021-7.

## Introduction

Lipoprotein (a) (Lp(a)) is a complex of an low-density lipoprotein-like (LDL-like) particle that contains an apolipoprotein B100 (apoB100) molecule and a glycoprotein apolipoprotein(a) (apo (a)), which linked by a disulfide bridge [1]. Plasma concentrations of Lp(a) range from 1 mg /dl to 300 mg /dl in human [2] and are rarely affected by gender, age, lifestyle and food intake [3]. At present, abundant studies have demonstrated that increased Lp(a) is a risk factor for cardiovascular disease especially coronary artery disease and atherosclerosis [4–8]. The relationship between Lp(a) and diabetes remains controversial. Some studies suggested that high Lp(a) levels could increase diabetes risk [9], some investigations indicated that Lp(a) had no association with diabetes [10, 11], but in recent years, increasing studies demonstrated that T2DM patients had lower Lp(a) levels [6, 12–14]. What’s more, Lp(a) is also associated with other metabolic diseases, such as non-alcoholic fatty liver disease (NAFLD) and metabolic syndrome (Mets) [15, 16].

Obese patients are often accompanied by glucose and lipid metabolism disorders. Lp(a), as an indicator of lipid metabolism, often changes in obese people. What is more, metabolism disorders can further affect hormone levels. However, there are few studies on Lp(a) and the relationship between Lp(a) and hormones in obese patients. By exploring the association between variation of Lp(a) and various hormone levels in overweight/obese people, we found serum testosterone levels is negatively associated with Lp(a) levels, which maybe provide a new idea for discovering the mechanism between Lp(a) and other diseases.

## Materials and methods

### Study design and subjects

A total of 410 overweight/obese participants were included in this cross-sectional study from January 2017 to November 2020 at the Medical Nutrition and Weight Loss Center of Beijing Chao-Yang Hospital, Capital Medical University. All of them were asked about their histories of diseases and drugs and received laboratory tests. The inclusion criteria were as follows: age between 15 and 65 years and BMI ≥ 25 kg/m^2^. The patients with cardiovascular disease, infectious disease, severe hepatic or renal insufficiency, systemic inflammatory disease, cancer, use of hypoglycemic or lipid-lowering drugs were excluded. All subjects were divided into three groups based on the Lp(a) levels: the lower Lp(a) group (Lp(a) levels < 30 mg/dl), the moderate Lp(a) group (Lp(a) levels between 30 mg/dl and 120 mg/dl) and the higher Lp(a) group (Lp(a) levels > 120 mg/dl) [3].

All participants provided written informed consent. The protocol of this study was approved by the Ethics Committee of Beijing Chao-Yang Hospital, Capital Medical University.

### Clinical and biochemical measurements

For each patient, the following demographic data were collected: gender, age, body height and weight. After an overnight fasting, venous blood samples of every subject were obtained for further measurement at the central chemistry laboratory of the Beijing Chao-Yang Hospital, Capital Medical University. Total cholesterol (TC), triglyceride (TG), low-density lipoprotein cholesterol (LDL-C), high-density lipoprotein cholesterol (HDL-C) and Lp(a) were analyzed by colorimetric enzymatic assays using a Dade Behring Dimension RXL autoanalyzer (Dade Behring Diagnostics, Marburg, Germany). Fasting blood glucose (FBG) was estimated with glucose oxidase method using Hitachi 747 and fasting insulin (FINS) was estimated by chemiluminescence immunoassay using a Centaur XP immunoassay system (Siemens Healthcare Diagnostics, Germany). Growth hormone (GH), testosterone (TES), follicle-stimulating hormone (HFSH), prolactin (PRL), luteinizing hormone (LH) and estradiol (E2) were measured with chemiluminescence immunoassay method using Access 2 immunoassay system (Beckman Coulter, Inc., Brea, CA, USA). Body mass index (BMI) was calculated using following formula: body weight (kg)/[height(m)]^2^. Homoeostasis model assessment of insulin resistance (HOMA-IR) was used to estimate insulin resistance, which calculated using the following formula: HOMA-IR = FBG (mmol/L) × FINS (mU/L) / 22.5.

### Statistical analysis

All statistical analyses were performed using software SPSS (version 26.0, SPSS Inc., Chicago, IL, USA). For the analysis of differences among groups, the variables of normal distribution, represented by mean ± standard deviation, were assessed with one-way ANOVA and Bonferroni was selected for post-hoc comparison, while non-normally distributed data, expressed by medians with interquartile range, were evaluated with Kruskal-Wallis analysis and Bonferroni analysis was used for correction. The proportions were analyzed by chi-square tests. Spearman’s rank correlation was used to assess the relationship between Lp(a) and other indices. Multiple linear regression analysis (enter method) was used to analyze whether the variables were associated with Lp(a) after adjusting for potential confounding factors. All tests were two-tailed, and *P <* 0.05 were considered statistically significant.

## Results

### Characteristics of overweight/obese patients with different levels of Lp(a)

Clinical data pertaining to the metabolism of the overweight/obese patients were presented in Table 1. All subjects were divided into three groups based on the Lp(a) levels: the lower Lp(a) group contained 137 participants, the moderate Lp(a) group contained 137 participants and the higher Lp(a) group contained 136 participants. The levels of LDL-C, TC, GH and TES were increased in the higher Lp(a) group in contrast with the lower Lp(a) group. Moreover, significant differences in LDL-C and TES levels were observed between the higher Lp(a) group and the moderate Lp(a) group. Additionally, other hormones such as HFSH, LH, PRL and E2 did not show difference statistically.

**Table 1:** Characteristics of Chinese obese participants with various levels of Lp(a)

Variables	All Study Subjects			*P* value
	Lower Lp(a)	Moderate Lp(a)	Higher Lp(a)	
	(*n* = 137)	(*n* = 137)	(*n* = 136)	
Age (years)	31 ± 0.79	32 ± 0.76	33 ± 0.86	0.271
Gender-M/F	59 / 78	62 / 75	51 / 85	0.408
BMI (kg/m^2^)	38.82 ± 0.55	40.20 ± 0.67	38.35 ± 0.65	0.097
TC (mmol/L)	4.73 ± 0.09	4.78 ± 0.08	4.97 ± 0.09 *	0.016*
TG (mmol/L)	1.77 (1.20–2.68)	1.67 (1.22–2.50)	1.59 (1.32–2.00)	0.114
HDL-C (mmol/L)	1.03 ± 0.02	1.01 ± 0.02	1.07 ± 0.02	0.076
LDL-C (mmol/L)	3.00 ± 0.07	3.06 ± 0.06	3.24 ± 0.07 *#	< 0.001**
FBG (mmol/L)	5.71 (5.14–7.53)	5.80 (5.39–7.13)	5.75 (5.32–6.81)	0.591
FINS (uIU/ml)	25.36 (18.98–41.34)	26.91 (17.89–40.39)	24.13 (17.39–35.83)	0.319
GH (ng/ml)	0.03 (0.02–0.10)	0.04 (0.02–0.12)	0.05 (0.03–0.20) **	0.006*
TES (nmol/L)	3.24 (1.95–9.30)	3.10 (1.78–8.36)	2.21 (1.54–7.18) **#	0.003*
HFSH (mIU/ml)	5.21 (3.71–6.94)	5.10 (3.82–6.60)	5.17 (2.99–6.96)	0.885
LH (mIU/ml)	5.02 (3.19–7.50)	5.03 (3.36–7.07)	5.05 (3.20–7.84)	0.979
PRL (mIU/ml)	253.49 (188.79–347.58)	301.51 (190.80–400.54)	257.92 (182.92–358.71)	0.205
E2(pmol/L)	208.18 (143.13–281.61)	181.07 (133.48–292.58)	205.49 (147.40–314.56)	0.277
HOMA-IR	7.12 (4.85–12.89)	7.74 (4.83–12.01)	6.84 (4.49–10.42)	0.355

Normally distributed variables were expressed as mean ± standard deviation (SD), while variables with non-normal distributed were expressed as medians with first quartile to third quartile. *Lp(a)* lipoprotein (a); *BMI* body mass index, *TC* total cholesterol, *TG* triglyceride, *HDL-C* high-density lipoprotein cholesterol, *LDL-C* low-density lipoprotein cholesterol, *FBG* fasting blood glucose, *FINS* fasting insulin, *GH* growth hormone, *TES* testosterone, *HFSH* follicle-stimulating hormone, *LH* luteinizing hormone, *PRL* prolactin, *E*2 estradiol, *HOMA-IR* homeostasis model assessment of insulin resistance. Compared with the lower Lp(a) group, * *P* < 0.05, ** *P* < 0.001. Compared with the moderated Lp(a) group, # *P* < 0.05

Clinical metabolic characteristics of the gender subgroups were presented in Supplemental Table 1 and Supplemental Table 2. In male subgroup, the higher Lp(a) group showed significantly higher GH and LDL-C concentration and significantly decreased TES concentration relative to the lower Lp(a) group, as well as statistical difference in GH levels between the moderate group and the higher group were observed. In female subgroup, the levels of LDL-C were higher and the levels of TES were lower in the higher Lp(a) group compared with the lower Lp(a) group. Interestingly, the higher Lp(a) group had decreased TES concentration compared with the moderate Lp(a) group. Other parameters did not show difference among three groups in gender subgroups.

### Correlation analysis between Lp(a) and other parameters

Table 2 showed that Lp(a) was positively correlated with GH (*r* = 0.139, *P* < 0.05) and negatively correlated with TG (*r* = − 0.142, *P* < 0.05) and TES (*r* = − 0.190, *P* < 0.001) in all participants. Additionally, the negative association between Lp(a) and TES was also observed in the male and female groups respectively (Table 2). Furthermore, there was a significant positive correlation between HDL-C, LDL-C, GH, E2 and Lp(a) while negative correlation between Lp(a) and TG in the male group. We also found Lp(a) was positively correlated with TC and LDL-C in the female group.

**Table 2:** Association between Lp(a) and laboratory indices in all participants, male group and female group, respectively

	Lp(a)					
	All		Male		Female	
	*r*	*P* value	*r*	*P* value	*r*	*P* value
Age (years)	0.041	0.409	0.057	0.457	0.058	0.373
BMI (kg/m^2^)	−0.019	0.697	0.076	0.322	−0.052	0.421
TC (mmol/L)	0.081	0.347	0.095	0.213	0.176	0.006*
TG (mmol/L)	−0.142	0.004*	−0.178	0.020*	−0.090	0.168
HDL-C (mmol/L)	−0.016	0.855	0.175	0.022*	0.060	0.360
LDL-C (mmol/L)	0.132	0.123	0.183	0.016*	0.228	< 0.001**
FBG (mmol/L)	−0.039	0.443	−0.005	0.953	−0.027	0.688
FINS (uIU/ml)	−0.069	0.174	0.073	0.349	−0.058	0.383
GH (ng/ml)	0.139	0.006*	0.223	0.004*	0.054	0.408
TES (nmol/L)	−0.190	< 0.001**	−0.206	0.007*	−0.201	0.002*
HFSH (mIU/ml)	−0.021	0.669	−0.085	0.269	−0.008	0.902
LH (mIU/ml)	0.016	0.743	0.009	0.910	−0.015	0.815
PRL (mIU/ml)	0.047	0.345	0.019	0.804	0.040	0.538
E2 (pmol/L)	0.063	0.210	0.175	0.025*	−0.021	0.749
HOMA-IR	−0.067	0.188	−0.046	0.557	−0.055	0.415

*Lp(a) *lipoprotein (a); *BMI* body mass index, *TC* total cholesterol, *TG* triglyceride, *HDL-C* high-density lipoprotein cholesterol, *LDL-C* low-density lipoprotein cholesterol, *FBG* fasting blood glucose, *FINS* fasting insulin, *GH* growth hormone, *TES* testosterone, *HFSH* follicle-stimulating hormone, *LH* luteinizing hormone, *PRL* prolactin, *E*2 estradiol, *HOMA-IR* homeostasis model assessment of insulin resistance. * *P* < 0.05, ** *P* < 0.001

### Multiple linear regression analysis between Lp(a) and other indices

To further assess the relationship between Lp(a) and other parameters, we conducted a multiple linear regression analysis on the significant variables found by Spearman’s rank correlation analysis or considered clinically relevant, including gender, age, BMI, TC, TG, HDL-C, LDL-C, TES and GH (Table 3). We found that TES levels were independently related with Lp(a) levels (***β*** = − 0.266, *P* < 0.05, 95% CI = − 0.451 to − 0.079) after adjustment for gender, age, BMI, TC, TG, HDL-C and LDL-C. This significant result was also observed in male and female participants, respectively (Supplemental Table 3 and Supplemental Table 4).

**Table 3:** Multiple linear regression between Lp(a) levels and other parameters in all participants

Variables	***β***	*P* value	95%CI
Age (years)	0.036	0.486	[−0.067, 0.141]
Gender-Female ^a^	−0.184	0.064	[−0.382, 0.011]
BMI (kg/m^2^)	−0.094	0.107	[−0.208. 0.020]
TC (mmol/L)	0.001	0.998	[−0.474, 0.475]
TG (mmol/L)	−0.154	0.123	[−0.349, 0.042]
HDL-C (mmol/L)	0.024	0.752	[−0.126, 0.174]
LDL-C (mmol/L)	0.161	0.446	[−0.261, 0.594]
GH (ng/ml)	0.037	0.455	[−0.061, 0.137]
TES (nmol/L)	−0.266	0.005*	[−0.451, − 0.079]

*BMI* body mass index, *TC* total cholesterol, *TG* triglyceride, *HDL-C* high-density lipoprotein cholesterol, *LDL-C* low-density lipoprotein cholesterol, *GH* growth hormone, *TES* testosterone, *CI* confidence interval. * *P* < 0.05, ^a^ male as control

## Discussion

The purpose of this study was to investigated the the relationship between Lp(a) and hormones in Chinese overweight/obese people. Our results showed that serum TC, LDL-C and GH levels were significantly increased in the higher Lp(a) group compared with the lower Lp(a) group, as well as the higher Lp(a) group had higher LDL-C levels compared with moderate groups. Despite comparable clinical parameters, the patients with an increased Lp(a) had lower levels of TES compared with the patients with a moderate and lower Lp(a). We also observed serum TES levels were significantly decreased with the increase of Lp(a) levels whether stratified by gender or not. Multiple regression analyses showed that the TES was independently related with Lp(a) levels after adjusting for confounding factors.

We found Lp(a) always had a significant association with lipid metabolism indices, such as LDL-C. These results reiterated the close relationship between Lp(a), an indicator of lipid metabolism, and other lipid metabolism parameters. As we all know, ApoB100 and apo(a) exist in a molar ratio of 1:1 in Lp(a) and apo(a) can be isolated from the LDL-like portion only by reductive cleavage [17]. What’s more, the main locus controlling Lp(a) concentration is the LPA gene [18]. Lp(a) biosynthesis includes the following processes: (i) transcription of LPA gene; (2) protein translation; (iii) motion through the secretory pathway; (iv) assembly of Lp(a) composition [1]. All of these are likely to be regulated to contribute to concentration of plasma Lp(a). The evidence for extracellular synthesis of apo(a) was found in several human studies, in part from circulating LDL, while evidence for cycling and recombination of apo(a) into Lp(a) has also been represented [19, 20].

In our study, we also found that TES and GH were associated with Lp(a) levels, particularly TES, which indicated hormones were responsible for the concentration of Lp(a). This finding was consistent with previous research, which serum Lp(a) concentration increased after 6 weeks in GH-deficient adults treated with recombinant GH [21]. Moreover, there was a study investigated the effect of TES on Lp(a). The finding reported inhibition of TES lead to increased Lp(a) levels [22]. It also has been reported that Lp(a) is related to bile acid content, body weight change and insulin change [23–26], but their mechanism has not been clarified. Our research further proofed that TES was negatively correlated with Lp(a) levels whatever in male or female people.

A previous study has reported that serum levels of Lp(a) are related to waist circumference in NAFLD patients with low prevalence of co-morbidities, which proved larger waists, where the thickness of subcutaneous fat exceeds visceral fat, are predictors of levels of serum Lp(a) [27]. Interestingly, waist circumference is inversely associated with total and free TES has been reported by previous investigators in community dwelling men [28]. These conclusions are consistent with our findings. A explanation for the link between Lp(a) and TES maybe come from inflammatory cytokines. It has been reported by earlier studies that IL-17 related chemokine (Eotaxin) is central to the amount of visceral fat and obesity was associated with increased IL-17A production in humans [29, 30]. What is more, testosterone suppresses proinflammatory and up regulates anti inflammatory cytokines [31, 32]. Also there was negative association between IL-17 and TES, indicating inflammation suppresses testosterone levels [33].

There are several controversial reports on the relationship between Lp(a) concentrations and metabolic diseases. It is well known that Lp(a) has an independent causal association with cardiovascular disease, which has been extensively demonstrated from various aspects. The results of the studies showed that Lp(a) concentration was associated dose-dependently with coronary heart disease risk, peripheral artery disease, heart failure, and lifespan [6], suggesting that Lp(a) is a potent harbinger of clinical cardiovascular disease [4]. Mendelian randomization analysis further confirmed the above conclusion [8]. However, the effects of Lp(a) on diabetes were not consistent, and it was currently more likely to think that low Lp(a) concentration was associated with diabetes [6, 12–14]. Nevertheless, the consensus that diabetes was a risk factor for cardiovascular disease contradicted the above research conclusion. So, we need to further explore the reasons behind it. Additionally, our study provides a basis for the changes of metabolic indicators in clinical patients. For the patients treated with TES, Lp(a) levels may change, leading to other metabolic disorders.

There are several limitations in our study. Firstly, this study is cross-sectional research which resulting to no causal relationship can be drawn. Therefore, prospective studies are needed to confirm the conclusion. Secondly, compared with prior research, our study had a relatively small sample size. Finally, sex hormone secretion including TES, HFSH, LH, PRL and E2 might be affected by the menstrual cycle in female. However, the present study was not standardized for the menstrual cycle.

## Conclusion

TES levels was negatively associated with Lp(a) levels in Chinese overweight/obese patients.

## Supplementary Information

**Additional file 1 Supplemental Table 1.** Characteristics of male participants with various levels of Lp(a). **Supplemental Table 2.** Characteristics of female participants with various levels of Lp(a). **Supplemental Table 3.** Multiple linear regression between Lp(a) levels and other parameters in male participants. **Supplemental Table 4.** Multiple linear regression between Lp(a) levels and other parameters in female participants.

## Data Availability

The datasets used and/or analysed during the current study are available from the corresponding author on reasonable request.

## Abbreviations

Lp(a): Lipoprotein (a)
BMI: Body mass index
TC: Total cholesterol
TG: Triglyceride
HDL-C: High-density lipoprotein cholesterol
LDL-C: Low-density lipoprotein cholesterol
FBG: Fasting blood glucose
FINS: Fasting insulin
GH: Growth hormone
TES: Testosterone
HFSH: Follicle-stimulating hormone
LH: Luteinizing hormone
PRL: Prolactin
E2: Estradiol
HOMA-IR: Homeostasis model assessment of insulin resistance
CI: Confidence interval
