# Supplementary material for: Study on the relationship between hormone and Lp(a) in Chinese overweight/obese patients
Source: BMC Endocr Disord. 2022 May 16;22:131. doi: 10.1186/s12902-022-01021-7 (PMC9109374; doi:10.1186/s12902-022-01021-7)
Supplement: Supplementary file 1 — Additional file 1 Supplemental Table 1. Characteristics of male participants with various levels of Lp(a). Supplemental Table 2. Characteristics of female participants with various levels of Lp(a). Supplemental Table 3. Multiple linear regression between Lp(a) levels and other parameters in male participants. Supplemental Table 4. Multiple linear regression between Lp(a) levels and other parameters in female participants. [file 12902_2022_1021_MOESM1_ESM.docx]

Supplemental Table 1 Characteristics of male participants with various levels of Lp(a).

|  | | | | |
| --- | --- | --- | --- | --- |
|  | Male Subjects | | |  |
|  | Lower Lp(a) | Moderate Lp(a) | Higher Lp(a) |  |
| Variables | (n=59) | (n=62) | (n=51) | P value |
| Age (years) | 33 ± 9.87 | 33 ± 9.87 | 35 ±11.05 | 0.405 |
| BMI (kg/㎡) | 39.48 ± 7.19 | 42.86 ± 9.04 | 40.55 ± 9.33 | 0.087 |
| TC (mmol/L) | 4.82 ± 1.05 | 4.87 ± 0.84 | 5.18 ± 1.17 | 0.138 |
| TG (mmol/L) | 2.08 (1.41-3.73) | 1.74 (1.23-2.64) | 1.79 (1.41-2.54) | 0.169 |
| HDL-C (mmol/L) | 0.95 ± 0.19 | 0.95 ± 0.18 | 1.01 ± 0.15 | 0.143 |
| LDL-C (mmol/L) | 3.02 ± 0.78 | 3.12 ± 0.62 | 3.43 ± 0.94* | **0.018** |
| FBG (mmol/L) | 5.85 (5.29-8.08) | 6.03 (5.50-8.16) | 6.05 (5.43-8.26) | 0.684 |
| FINS (uIU/ml) | 28.70 (17.66-48.42) | 31.86 (20.62-48.15) | 28.48 (17.96-39.66) | 0.418 |
| GH (ng/ml) | 0.03 (0.01-0.06) | 0.03 (0.02-0.06) | 0.05 (0.03-0.15) *# | **0.002** |
| TES (nmol/L) | 10.21 ± 3.76 | 9.18 ± 3.65 | 8.35 ± 3.41* | **0.028** |
| HFSH (mIU/ml) | 4.71 (3.51-6.36) | 4.66 (3.24-6.01) | 4.58 (2.89-6.93) | 0.866 |
| LH (mIU/ml) | 4.54 ± 1.88 | 4.47 ± 1.65 | 4.74 ± 2.31 | 0.758 |
| PRL (mIU/ml) | 209.96 (161.34-287.69) | 240.37 (166.73-354.68) | 229.19 (159.91-285.42) | 0.483 |
| E2(pmol/L) | 153.86 (123.31-206.45) | 158.83 (124.32-204.00) | 189.66 (146.41-249.01) | **0.027** |
| HOMA-IR | 8.24 (5.26-14.84) | 8.82 (6.32-14.83) | 7.93 (5.79-11.58) | 0.646 |

Normally distributed variables were expressed as mean ± standard deviation (SD), while variables with non-normal distributed were expressed as medians with first quartile to third quartile. Lp(a) - lipoprotein (a); BMI – body mass index; TC - total cholesterol; TG - triglyceride; HDL-C - high-density lipoprotein cholesterol; LDL-C - low-density lipoprotein cholesterol; FBG - fasting blood glucose; FINS - fasting insulin; GH - growth hormone; TES – testosterone; HFSH - follicle-stimulating hormone; LH - luteinizing hormone; PRL - prolactin; E2 - estradiol; HOMA-IR - homeostasis model assessment of insulin resistance. Compared with the low Lp（a）group, * P < 0.05. Compared with the moderated Lp(a) group, # P < 0.05.

Supplemental Table 2 Characteristics of female participants with various levels of Lp(a).

|  | | | | |
| --- | --- | --- | --- | --- |
|  | Female Subjects | | |  |
|  | Lower Lp(a) | Moderate Lp(a) | Higher Lp(a) |  |
| Variables | (n=78) | (n=75) | (n=85) | P value |
| Age (years) | 30 ± 8.59 | 31 ± 7.99 | 32 ± 9.20 | 0.489 |
| BMI (kg/㎡) | 38.32 ± 5.71 | 38.00 ± 5.98 | 37.04 ± 6.06 | 0.354 |
| TC (mmol/L) | 4.61 ± 0.91 | 4.74 ± 0.74 | 4.90 ± 0.65 | 0.065 |
| TG (mmol/L) | 1.66 (1.16-2.21) | 1.63 (1.19-2.23) | 1.50 (1.19-1.85) | 0.315 |
| HDL-C (mmol/L) | 1.09 ± 0.24 | 1.09 ± 0.20 | 1.12 ± 0.19 | 0.581 |
| LDL-C (mmol/L) | 2.90 ± 0.69 | 3.03 ± 0.57 | 3.18 ± 0.53* | **0.010** |
| FBG (mmol/L) | 5.61 (5.02-6.65) | 5.58 (5.12-6.34) | 5.53 (5.12-6.21) | 0.857 |
| FINS (uIU/ml) | 24.35 (19.30-36.51) | 23.30 (16.72-39.14) | 23.27 (17.27-34.43) | 0.649 |
| GH (ng/ml) | 0.05 (0.02-0.12) | 0.07 (0.03-0.27) | 0.06 (0.03-0.26) | 0.133 |
| TES (nmol/L) | 2.15 ± 0.99 | 2.02 ± 0.78 | 1.64 ± 0.59**# | **<0.001** |
| HFSH (mIU/ml) | 5.92 (3.88-7.13) | 5.44 (4.15-6.82) | 5.57 (3.81-7.12) | 0.855 |
| LH (mIU/ml) | 6.36 (3.31-9.04) | 5.96 (3.29-9.51) | 5.64 (3.52-9.15) | 0.928 |
| PRL (mIU/ml) | 271.52 (206.40-399.79) | 327.44 (248.84-449.52) | 296.73 (216.97-429.71) | 0.222 |
| E2(pmol/L) | 247.16 (191.00-359.07) | 237.72 (135.63-422.54) | 221.09 (148.84-431.50) | 0.661 |
| HOMA-IR | 6.34 (4.72-11.46) | 6.68 (4.02-10.42) | 6.02 (4.06-9.49) | 0.621 |

Normally distributed variables were expressed as mean ± standard deviation (SD), while variables with non-normal distributed were expressed as medians with first quartile to third quartile. Lp(a) - lipoprotein (a); BMI – body mass index; TC - total cholesterol; TG - triglyceride; HDL-C - high-density lipoprotein cholesterol; LDL-C - low-density lipoprotein cholesterol; FBG - fasting blood glucose; FINS - fasting insulin; GH - growth hormone; TES – testosterone; HFSH - follicle-stimulating hormone; LH - luteinizing hormone; PRL - prolactin; E2 - estradiol; HOMA-IR - homeostasis model assessment of insulin resistance. Compared with the low Lp（a）group, * P < 0.05, ** P < 0.001. Compared with the moderated Lp(a) group, # P < 0.05.

Supplemental Table 3 Multiple linear regression between Lp(a) levels and other parameters in male participants.

|  | | | |
| --- | --- | --- | --- |
| Variables | **β** | P value | 95% CI |
| Age (years) | 0.044 | 0.578 | [-0.089, 0.158] |
| BMI (kg/㎡) | -0.111 | 0.226 | [-0.209, 0.050] |
| TC (mmol/L) | -0.034 | 0.927 | [-0.590, 0.537] |
| TG (mmol/L) | -0.165 | 0.354 | [-0.322, 0.116] |
| HDL-C (mmol/L) | -0.017 | 0.858 | [-0.207, 0.173] |
| LDL-C (mmol/L) | 0.170 | 0.611 | [-0.381, 0.645] |
| GH (ng/ml) | 0.317 | **<0.001**** | [0.334, 0.913] |
| TES (nmol/L) | -0.230 | **0.009*** | [-0.407, -0.058] |
|  | | | |

BMI - body mass index; TC - total cholesterol; TG - triglyceride; HDL-C - high-density lipoprotein cholesterol; LDL-C - low-density lipoprotein cholesterol; GH - growth hormone; TES - testosterone; CI - confidence interval. * P < 0.05, ** P < 0.001.

Supplemental Table 4 Multiple linear regression between Lp(a) levels and other parameters in female participants.

|  | | | |
| --- | --- | --- | --- |
| Variables | **β** | P value | 95% CI |
| Age (years) | -0.064 | 0.380 | [-0.252, 0.096] |
| BMI (kg/㎡) | -0.043 | 0.538 | [-0.241, 0.126] |
| TC (mmol/L) | 0.127 | 0.662 | [-0.558, 0.876] |
| TG (mmol/L) | -0.200 | **0.032*** | [-0.762, -0.034] |
| HDL-C (mmol/L) | -0.005 | 0.962 | [-0.226, 0.215] |
| LDL-C (mmol/L) | 0.111 | 0.669 | [-0.503, 0.782] |
| GH (ng/ml) | -0.030 | 0.643 | [-0.139, 0.086] |
| TES (nmol/L) | -0.210 | **0.003*** | [-2.023, -0.406] |

BMI - body mass index; TC - total cholesterol; TG - triglyceride; HDL-C - high-density lipoprotein cholesterol; LDL-C - low-density lipoprotein cholesterol; GH - growth hormone; TES - testosterone; CI - confidence interval. * P < 0.05.
